# The Work of the Canadian Medical Service

**Published:** 1916-09-30

**Authors:** 


### THE CANADIAN CASUALTY SYSTEM.

### The Work of the Canadian Medical Service.

The hospital system of Canada differs essentially from that of the Mother Country in that it is not of organic growth, but has been devised and built up to meet the needs and minister to conditions incidental to a new and rapidly growing country. Maintained by Provincial or Federal Government subventions and possessed of a character altogether free from any taint of charity, the municipal infirmaries stand in much closer relation to the general life of the community than does any hospital at home; and they are regarded as much a civic possession as the electric light, the water, or the street-railway service.

Designed to meet the requirements of an expanding population under peace conditions, the war has subjected this hospital system to a severe and unprecedented strain. From the day when the first Dominion troops assembled at the camp at Valcartier in Quebec to the present hour, the Canadian Medical Service, recruited from the hospitals throughout Canada, has kept step with the battalions, and has maintained an unbroken connection from the trench in Flanders to the very shores of the Pacific eight thousand miles away, where the Esquimault Military Convalescent Hospital stands to receive its broken soldiers. All told, in training, in reserve, and at the Front, Canada has something approaching half a million men under arms. In this country alone—or perhaps, to be more exact, on this side of the Atlantic—she has equipped, has staffed, and is maintaining nearly thirty hospitals for the use of her troops; and while it has not been found possible in actual practice exclusively to reserve these hospitals for the use of Dominion casualties, they nevertheless remain Canadian in *personnel* and system, even though, as in some instances, little more than 8 per cent. of the patients are drawn from Canadian battalions.

The system employed in handling casualties has recently been set forth in an excellent chart issued by the Military Hospitals Commission, a Canadian organisation formed last year at the instance of the Prime Minister, Sir Robert Borden, for the purpose of supervising the hospital accommodation and military convalescent homes in Canada for officers, non-commissioned officers, and men invalided while on active service. In passing it may be recorded that the Commission has already done magnificent service, not only in the creation of a chain of convalescent hospitals throughout the country, but in the establishment of such special institutions as a vocational training centre (to which is linked a Provincial Employment Commission), several sanatoria for the treatment of tuberculosis, special hospitals for nerve cases and rheumatism, and a large central orthopœdic institution at Toronto. The activities of the Commission have even embraced a summer camp and a home for rest.

As will be seen, the system corresponds in general to, and is indeed founded upon, the routine employed in the British Medical Service, and can be considered characteristically Canadian only in the adroitness and energy with which a comparatively small peace establishment has been applied and extended to meet the exigencies of a situation unparalleled alike in the reach of its demands and the searching character of its experiences

The chart previously referred to exhibits the passage of a casualty from the firing-line to the point where re-junction with the battalion is achieved, or, following upon incapacity, a return to Canada and civil life. The casualty is received from the battalion in action and is passed to the regimental medical establishment, represented by the regimental medical officer and his assistants at work in some subjacent dug-out. A memorandum is there filled in giving the patient's regimental number and name, nature of wound or wounds, and the treatment administered. This memorandum is secured to the person of the casualty, who is thereafter passed down to the field ambulance. There the case is entered in the admission and discharge book—a procedure which is followed throughout all the future movements of the casualty—additional notes are made on the memorandum, and a report with a diagnosis is made to the base. From the field ambulance the casualty is conveyed to the casualty clearing station, where a brief "history" is made out and passed with the case to the stationary or general hospital. At this stage, if the injury is slight the patient in due course is moved up to the convalescent depôt, and thence to the Front again. If, however, the case is more serious the casualty crosses the Channel into the primary hospital, and the admission is reported to the Canadian Record Office. From the primary hospital the patient in the fulness of time goes to the Canadian Convalescent Hospital, where, when quite fit, he is passed into his reserve battalion, to continue his regular training and finally return to the Front in a reinforcing draft. If not quite fit, he is either boarded for return to Canada, or scheduled for light duty, or is sent to undergo a course of physical exercise and training for hardening-up prior to passing into his reserve battalion.

The record of all these movements is centred in the Canadian Record Office in London. The base hospital sends to London with diagnoses a daily nominal roll of admissions and discharges at the field ambulance, casualty clearing station, stationary hospital, and convalescent depôt; and similar daily reports are made by the primary hospital and the Canadian Convalescent Hospital. All these reports are combined by the Record Office in a daily Canadian casualty list. From this list the Director of Medical Services, Canadians, makes a card for each casualty when he first appears in a hospital. This card shows name, regimental number, corps or unit, diagnosis, hospital, and date admitted. The hospital and date admitted are posted on the card at each succeeding admission, and the death is posted if it supervenes. Finally, when the casualty is discharged from medical care the fact is noted.

All admission and discharge books from the field ambulance to convalescent hospital, together with the medical case-sheets, pass when three months old into the custody of the National Research Society, for the use of the Medical Statistics Bureau of the War Office.

The medical-history sheet of each Canadian is usually begun in Canada and forwarded with the soldier to England. If this has not been done, it is begun on his arrival in England, and it follows the soldier so long as he is in England, his medical history being posted on the sheet as it develops. When he proceeds to the seat of war the sheet is forwarded to the Canadian Record Office for safe custody; and when the soldier returns to England as a casualty the original sheet is forwarded to the hospital he enters, in order to connect with him. It follows him wherever he goes, and receives additional posting as necessary.

